# Structural Basis of the Interaction between Human Axin2 and SIAH1 in the Wnt/β-Catenin Signaling Pathway

**DOI:** 10.3390/biom13040647

**Published:** 2023-04-04

**Authors:** Lianqi Chen, Yan-Ping Liu, Li-Fei Tian, Mingzhou Li, Shuyu Yang, Song Wang, Wenqing Xu, Xiao-Xue Yan

**Affiliations:** 1 National Laboratory of Biomacromolecules, CAS Center for Excellence in Biomacromolecules, Institute of Biophysics, Chinese Academy of Sciences, Beijing 100101, China; 2 College of Life Sciences, University of Chinese Academy of Sciences, Beijing 100049, China; 3 School of Life Science and Technology, ShanghaiTech University, Shanghai 201210, China

**Keywords:** Wnt signaling pathway, Axis inhibition protein Axin2, E3 ubiquitin-protein ligase SIAH1, GSK3-binding site, X-ray crystal structure

## Abstract

The scaffolding protein Axin is an important regulator of the Wnt signaling pathway, and its dysfunction is closely related to carcinogenesis. Axin could affect the assembly and dissociation of the β-catenin destruction complex. It can be regulated by phosphorylation, poly-ADP-ribosylation, and ubiquitination. The E3 ubiquitin ligase SIAH1 participates in the Wnt pathway by targeting various components for degradation. SIAH1 is also implicated in the regulation of Axin2 degradation, but the specific mechanism remains unclear. Here, we verified that the Axin2-GSK3 binding domain (GBD) was sufficient for SIAH1 binding by the GST pull-down assay. Our crystal structure of the Axin2/SIAH1 complex at 2.53 Å resolution reveals that one Axin2 molecule binds to one SIAH1 molecule via its GBD. These interactions critically depend on a highly conserved peptide _361_**EMTPVEPA**_368_ within the Axin2-GBD, which forms a loop and binds to a deep groove formed by β1, β2, and β3 of SIAH1 by the N-terminal hydrophilic amino acids Arg361 and Thr363 and the C-terminal VxP motif. The novel binding mode indicates a promising drug-binding site for regulating Wnt/β-catenin signaling.

## 1. Introduction

The Wnt/β-catenin signaling pathway plays a central role in embryonic development and adult tissue homeostasis [1], and its dysfunction is tightly associated with many cancers, including colorectal, stomach, and breast cancer. In recent years, researchers have found that the Wnt signaling pathway regulates the maintenance of hematopoietic stem cells, neural stem cells, embryonic stem cells, and tumor stem cells [2,3]. Further study on the regulatory mechanism of the Wnt signaling pathway is of great significance for the treatment of various human diseases [4,5].

β-Catenin is the central effector of the canonical Wnt signaling pathway, and it is essential for the transcription of Wnt target genes in the nucleus [6]. In the absence of Wnt, free cytosolic β-catenin proteins are constantly earmarked for degradation by the β-catenin destruction complex in the cytoplasm and this prevents β-catenin from accumulating in the cytoplasm and nucleus, leading to repression of Wnt target genes. The presence of the Wnt signal leads to the inhibition of the β-catenin destruction complex, accumulation of cytoplasmic/nuclear β-catenin, and subsequent transactivation of Wnt target genes.

Axis inhibition protein (Axin) is a core component of the β-catenin destruction complex, in which it acts as the scaffolding protein using its discrete domains to interact with β-catenin, CK1, GSK3, and Dvl. It coordinates the sequential phosphorylation of β-catenin. β-Catenin phosphorylation at Ser45 by Axin-bound CK1α is the crucial priming step of sequential phosphorylation [7]. Within this complex, Axin is the key limiting factor; post-transcription modifications of Axin such as phosphorylation, poly-ADP-ribosylation (PARylation), and ubiquitination can affect its stabilization and interactions with its partners and influence phosphorylated and ubiquitination degradation of β-catenin [8,9].

Axin is ubiquitous in organisms and plays an important regulatory role in physiological processes such as embryonic development, tumor formation, and apoptosis. It is a main tumor inhibitor as well [10]. Axin1 and Axin2 are two members of the Axin family in humans [11]. Studies have shown that Axin1 and Axin2 perform the same function in the Wnt signaling pathway [8,12]. Moreover, Axin2 has been shown to partially replace the function of Axin1 during mouse embryonic development [13,14]. However, their distribution in cells differs, which means that Axin2 cannot always replace Axin1 to perform certain functions, and they have their irreplaceable roles [15,16]. Activation of the Wnt pathway can induce Axin2 protein expression, and Axin2 may provide a negative feedback loop for the downregulation of Wnt signaling, in the case of excessive cytoplasmic β-catenin accumulation [17]. In hepatoma cells, inhibition of Axin2 expression causes the abnormal activation of the Wnt/β-catenin signaling pathway and promotes the abnormal expression of transcription activator STAT3, which promotes the occurrence of tumors, revealing that Axin2 gene expression may be involved in the regulation of hepatocarcinogenesis [18]. Axin1 and Axin2 are potential drug targets for the therapeutic treatment of cancer [19]. The Axin family is involved in a variety of biological processes, and the expression and activity of Axin proteins are tightly regulated. Therefore, it is important to uncover the structural basis of the function of Axin1 and Axin2 in different biological processes for the development of highly effective drug targets.

Seven in absentia homolog (SIAH) is an evolutionarily conserved E3 ubiquitin ligase, which restricts its activity by self-ubiquitination, and it is an important regulator of pathways activated under hypoxic conditions [20]. It is also involved in stress, oxidative, DNA damage, and inflammatory responses [21]. SIAH1 is closely related to the development of human cancer, and is abnormal in breast, hepatoma, and prostate cancer cells [22]. Thus, it is expected to become a new cancer drug target. SIAH1 participates in the Wnt pathway by targeting various components to degradation, including competing with GSK3β for its substrate-binding domain (SBD) interaction with the VxP motif of Axin1, leading to the disassembly of the β-catenin destruction complex and ubiquitin-induced degradation of Axin1 [23]. SAIH-binding proteins all contain the conserved VxP motif, and the VxP motif of Axin is located in its GSK3-binding domain. SIAH1-dependent Axin1 ubiquitination degradation disrupts the assembly of the β-catenin destruction complex in the cytoplasm, which is a key rate-limiting step for maintaining the Wnt/β-catenin signal. Axin2 in the Wnt signaling pathway is also regulated by SIAH1 E3 ubiquitin ligase [23]. However, the specific molecular basis for the recognition of Axin2 by SIAH1 remains unclear.

Here, we showed that the Axin2-GBD is sufficient for SIAH1 binding. Our work revealed how the SIAH1-SBD recognizes and binds to the Axin2-GBD, indicating a novel binding mode for SIAH and Axin. Wnt-induced Axin2 is a key negative feedback regulator of the Wnt signaling pathway. This crystal structure of the Axin2/SIAH1 complex explains the pattern of Axin2 recognition by SIAH1 and enhances the role of Axin in the Wnt signaling regulation. This has important implications for designing inhibitors of the Axin2 and SIAH1 interactions.

## 2. Materials and Methods

### 2.1. Protein Cloning, Expression, and Purification

The full length *SIAH1* (GenBank: NM_003031) and *AXIN2* (GenBank: NM_004655.4) were amplified from Hela cDNA and cloned into a pET28a vector (Novagen, Beijing, China) with a TEV cleave site at the N-terminal. Mutants were introduced into SIAH1-SBD_89–282_ by QuikChange Lightening Site-Directed Mutagenesis Kit (Agilent Technologies, Santa Clara, CA, USA). SIAH1-SBD constructs were transformed into BL21(DE3) for protein expression. Cell cultures were induced with 0.2 mM isopropyl β-D-1-thiogalactopyranoside (IPTG) for 16 h at 20 °C when OD600 reached 0.8. Cell pellets were sonicated in lysis buffer (50 mM Tris-HCl pH8.0, 500 mM NaCl, 20 mM imidazole, 5 mM β-mercaptoethanol). After configuration, the SIAH1-SBD in supernatant was purified in lysis buffer (20 mM Tris-HCl pH8.0, 500 mM NaCl, 2 mM DTT) by the Ni-NTA affinity column, His-tag cleavage by TEV protease, Ni-NTA affinity column, HiTrap Q column, and Superdex 200 increase 10/300 GL column (GE Healthcare, Chicago, IL, USA). Purified protein samples were concentrated by ultrafiltration in 20 mM Tris-HCl pH8.0, 200 mM NaCl, 2 mM DTT, flash-frozen in liquid nitrogen, and stored at −80 °C.

Axin2 gene fragments were sub-cloned into pGEX-6p-1 vectors, including the N-terminal PreScission protease site for GST tag removal. The constructs transformed into BL21 (DE3) and cultured in LB at 37 °C to OD600 ~ 0.8. Cell cultures were induced with 0.2 mM IPTG at 16 °C for 18 h. The harvested bacteria were resuspended on ice and homogenized via sonication. After configuration (26,000 g for 1 h at 4 °C), the protein in the supernatant was purified in lysis buffer (20 mM Tris-HCl pH7.5, 500 mM NaCl, 2 mM DTT) by the glutathione sepharose 4B resin (GE Healthcare, Chicago, IL, USA) for use. Additionally, the GST tag was removed on the GST column by incubating with PreScission protease at 4 °C overnight after being washed with lysis buffer. The eluted proteins were purified by Superdex 75 increase 10/300 GL column (GE Healthcare, Chicago, IL, USA) using buffer containing 20 mM Tris-HCl pH8.0, 100 mM NaCl, 2 mM DTT. Purified protein samples were concentrated and flash-frozen in liquid nitrogen, stored at −80 °C.

### 2.2. Crystallization and Data Collection

For the crystallization attempt, 10 mg/mL of SIAH1-SBD protein was mixed with various constructs of Axin2 at a molar ratio of 1:5, then incubated on ice for 2 h, centrifugated for 10 min at 13,000 rpm, and screened for crystal growth. Crystal screening kits were Hampton Research’s Index, Crystal Screen I/II/Lite, PEGRx I/II, PEG/Ion I/II, MembFac, Natrix, and SaltRx I/II. The screening methods were selected by a hanging drop method and sitting drop method; 1.5 μL protein sample mixed with 1.5 μL pool solution was screened by hanging drop method, and 1 μL protein solution mixed with 1 μL pool solution was screened by sitting drop method. Crystal growth plates were placed at 20 °C and 4 °C for culture.

After the crystal growth droplet was placed in cold storage at 4 °C for 2 weeks, we were only able to obtain crystals with the Axin2 (residues 356–376) construct by the sitting drop vapor diffusion method at 4 °C from the Hampton PEGRx 1 solution 39 (0.1 M Bis-Tris propane pH9.0, 30% PEG6000). However, crystals should not be duplicated. Detergents and additives were screened by adding 0.1 M Bis-Tris propane pH 9.0 and 30% PEG6000 as a growth liquid. The screening system was 1.5 μL protein samples, 1.5 μL pool solution, 0.3 μL Additive/Detergent, and they were grown at 4 °C. Finally, the Additive 72 (5% *w*/*v* n-Dodecyl-β-D-maltoside) could be used to stabilize crystal growth.

Axin2/SIAH1 complex crystals were initially obtained in sheets and clusters without thickness or fragility, and were not suitable for crystallographic collection. To obtain high-quality crystals, the protein concentrations, buffer pH, and precipitator concentrations were individually optimized for Axin2/SIAH1 crystallization conditions, and the best crystals were continuously crushed and seeded into the next optimized crystal growth droplet. Crystals suitable for X-ray diffraction were grown in 0.1 M Bis-tris propane pH8.4, 24% PEG6000 using hanging drop vapor diffusion method at 4 °C by seeding. The crystals were frozen in a cryoprotective solution containing 0.1 M Bis-tris propane pH8.4, 24% PEG6000, 18% glycerol. X-ray diffraction data were collected on the beamline BL19U1 (λ = 0.979 Å) at Shanghai Synchrotron Radiation Facility (SSRF), and integrated and scaled using HKL3000 software [24].

### 2.3. Structure Determination and Refinement

The structure of the Axin2/SIAH1 complex was solved by a molecular replacement method using PHASER [25] in the CCP4 suite with one monomer of SIAH1 (PDB 4CA1) [26]. Iterative cycles of refinement and manual model building were carried out with PHENIX refinement [27] and COOT [28] at 29~2.53 Å. Structural superposition of Axin2/SIAH1 and Axin1/SIAH1 were performed based on the Cα’s of SIAH1-SBD. All structural images were generated using PyMOL [29]. The data collection and refinement statistics are summarized in Table 1.

### 2.4. GST Pull-Down Assays

Human Axin2 constructs were expressed and purified as described. A total of 20 uM SIAH of fragments, 5 uM of wild-type GST-Axin2 fragments, and 20 μL glutathione sepharose 4B beads (GE Healthcare, Chicago, IL, USA) were mixed in 100 μL of pull-down buffer containing 20 mM Tris-HCl pH7.2, 300 mM NaCl, and 1 mM DTT. The mixed samples were incubated at room temperature for 2 h, followed by washing the beads with the pull-down buffer three times. During each wash, 200 μL of pull-down buffer was added to each sample and incubated at room temperature for 5 min before centrifugation and removal of supernatant. After washing, the resin was boiled and analyzed by SDS-PAGE.

### 2.5. Western Blotting

Samples were separated by SDS-PAGE gel, and transferred to PVDF membranes using the Trans-Blot Turbo Transfer system (Bio-Rad, Hercules, CA, USA), and probed with antibodies Anti-6X His-tag (ab1187; Abcam, Cambridge, UK). Blots were developed by ECL (Bio-Rad, Hercules, CA, USA).

### 2.6. Biolayer Interferometry (BLI) Assays

The binding of Axin2 with SIAH1 was measured by BLI using Octet Red96 system (FerteBio, Fremont, CA, USA). GST, GST-Axin2-GBD, or GST-Axin2-GBD deletions were loaded onto GST biosensors (FerteBio, Fremont, CA, USA,) and GST biosensors were then quenched with GST to block the free sites of the biosensors. The biosensors were dipped into SIAH1-SBD solutions for the binding measurements. The concentration gradients of SIAH1-SBD used in these BLI assays were 0.03 μM, 0.1 μM, 0.3 μM, 1 μM, 3 μM, 10 μM, or 30 μM. The interference patterns from the free-GST-immobilized biosensors with the concentration gradients of SIAH1-SBD were analyzed as controls.

### 2.7. Isothermal Titration Calorimetry (ITC)

The binding affinities of SIAH1-SBD with Axin2_356–376_ (wild-type and mutants) were measured at 25 °C using ITC200 calorimeters (GE life science and MicroCal, Malvern, UK). All proteins were dialyzed against 20 mM Tris-HCl pH8.0, 100 mM NaCl and degassed. A total of 2 μL of 330 μM Axin2 peptide was injected 20 times into 300 μL of 30 μM SIAH1 protein with 4 min intervals. Data were analyzed using Origin software (Version 7.0). A single binding site model for SIAH1/Axin2 gave the best fit to the data. Errors are given as SD of the fit from the original data points.

## 3. Results and Discussion

### 3.1. Biochemical Characterization of Axin2/SIAH1 Interaction

**Figure 1:**
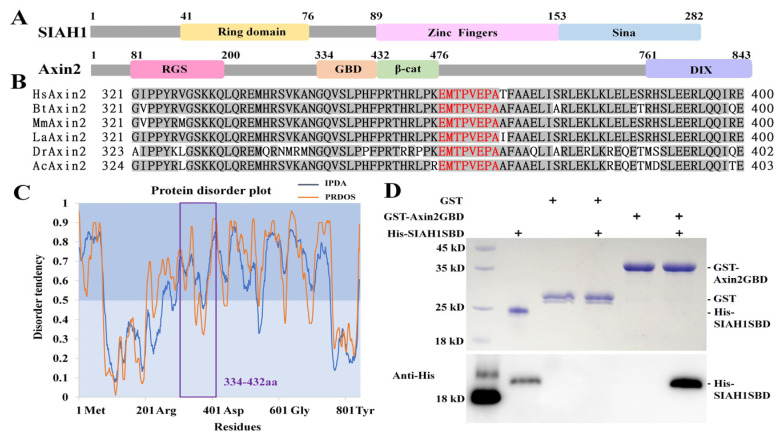
Biochemical characterization of the Axin2/SIAH1 interaction. (**A**) Schematic diagram of the human Axin2 and SIAH1 conserved domains. (**B**) Sequence alignment of Axin2-GBD indicated it is conserved in vertebrate evolution, in which the highly conserved peptide, including the VxP motif, are shown in red. Abbreviations: Hs, *Homo sapiens*; Bt, *Bos taurus*; Mm, *Mus musculus*; La, *Loxodonta africanar*; Dr, *Danio rerio*; Ac, *Anolis carolinensis*. (**C**) The protein disorder tendency prediction of the full length human Axin2, using the GeneSilico MetaDisorder server (http://iimcb.genesilico.pl/metadisorder/ accessed on 21 March 2023). The X-axis represents residues 1–801 of the full-length human Axin2 protein, whereas the Y-axis is the disorder tendency score for each residue in the context of the Axin2 sequence. The regions with the disorder tendency higher than 0.5 have a high tendency to be structurally disordered. Two different prediction methods, IPDA and PRDOS, are shown in blue and orange, respectively. (**D**) GST pull-down assays of the Axin2-GBD/SIAH1-SBD interaction by using the SDS-PAGE and Western blot.

Axin and SIAH proteins are highly conserved in vertebrate evolution. SIAH1 contains the conserved N-terminal ring domain and C-terminal domain (Figure 1A). Given that the full-length protein is poorly soluble and shows serious precipitation during expression and purification, the SBD of SIAH1 was selected for this work. SIAH1-SBD removed the N-terminal ring domain, leaving a total of 194 amino acid residues from 89 residues to 282 residues. Axin2 contains a conserved N-terminal RGS domain and C-terminal DIX domain (Figure 1A) Except for the N-terminal RGS domain and the C-terminal DIX domain, most of the other regions of Axin2 are irregular coil-coiled (Figure 1A,C), so it often binds to target proteins as small fragments [30,31,32,33,34]. Since SIAH1 can recognize the GBD regions of Axin2 [23], the Axin2-GBD with a GST-tag was first constructed to verify the interaction. Axin2-GBD is composed of 99 amino acids (334–432 residues) and is evolutionarily conserved (Figure 1B). By the SDS-PAGE and Western analysis, GST pull-down assays showed that the Axin2-GBD was sufficient to interact with the SBD of SIAH1, but their interaction forces were not particularly strong (Figure 1D), which is consistent with the in vivo assays [23].

**Figure 2:**
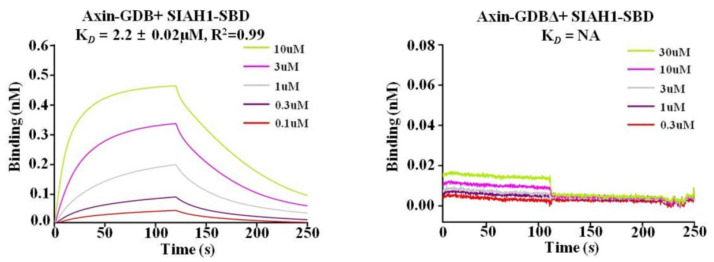
Binding affinities of Axin-GDB (**left**) or Axin-GDBΔ (**right**) with SIAH1-SBD, as measured by BLI assays.

Subsequently, we measured the affinity between Axin2-GBD and SIAH1-SBD using the BLI assay. The results revealed a micromolar interaction with Axin-GDB and SIAH1-SBD (*K_D_* of ~ 2.2 μM, Figure 2 left), but this binding affinity was not detected for the Axin-GDBΔ (deletion without _361_**EMTPVEPA**_368_, Figure 2 right), confirming the importance of this VxP motif.

### 3.2. Crystal Structure of the Axin2/SIAH1 Complex

The structure (PDB ID code 8HEO) was solved at 2.53Ǻ. There were two SIAH1-SBD molecules in each asymmetric unit of the crystal structure of the Axin2/SIAH1 complex: one with an Axin2 peptide, and in the other, the Axin2 peptide was not constructed because of its poor electron density. The main crystallographic parameters are reported in Table 1.

**Figure 3:**
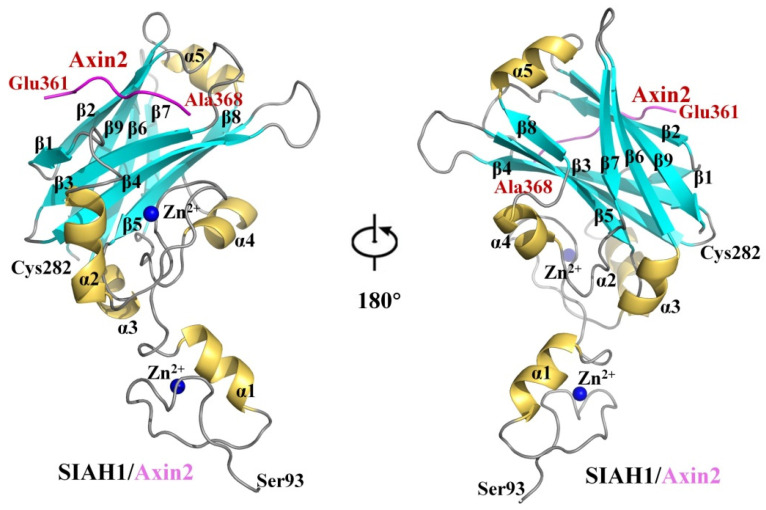
Overall structure of the Axin2/SIAH1 complex. Cartoon representation of the Axin2/SIAH1 complex with secondary structural elements labeled (α: α-helix; β: β-strand); β-strand is represented in cyan, α-helix is represented in yellow; Axin2-GBD are shown in purple.

In our Axin2/SIAH1 complex structure, there are two zinc fingers and nine β-strands located at the N-terminal domain and C-terminal SINA domain of SIAH1, respectively (Figure 3). Cys98, Cys105, Cys121, and His117 formed a coordination bond with a Zn^2+^, forming the first zinc finger motif. His147, Cys128, Cys135, and His152 formed coordination bonds with another Zn^2+^, forming a second zinc finger motif. Zinc finger structures are mostly found in transcription factors that interact with DNA, and they can regulate interactions between characteristic proteins [35]. A zinc finger motif was also located in the N-terminal RING domain of SIAH, which is mainly involved in the activity of ubiquitin ligase [36]. However, the specific function of the zinc finger motif located in the SIAH-SBD remains unclear. The SIAH1-SINA domain was mainly composed of nine β-strands. In particular, β3 (177–184aa), β4 (187–196aa), β5 (204–211aa), and β8 (257–260aa) were close to one another, forming a β-sheet. β2 (162–168aa), β9 (272–281aa), β6 (221–228aa), and β7 (233–238aa) formed another β-sheet; β1 (154–159aa) was parallel to β2, which connected the N-terminal zinc finger motif with the SINA domain at the C-terminal. There were short α-helix structures between β5, β6, β7, β8, β8, and β9 (Figure 3).

**Figure 4:**
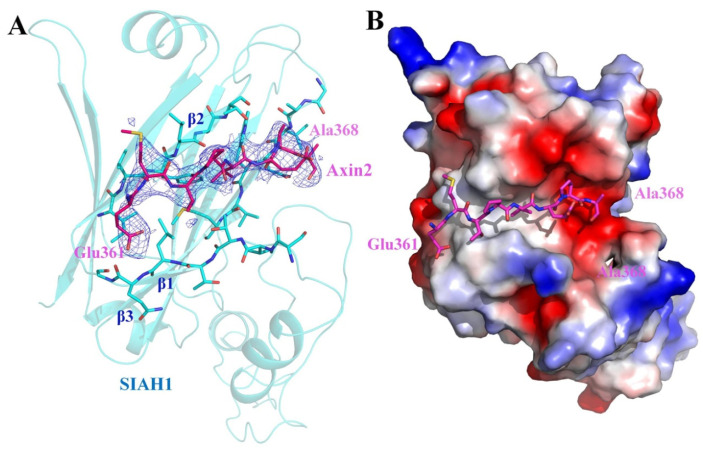
Axin2 peptide_361–368_ binds to the groove formed by β1, β2, and β3 of SIAH1. (**A**) The 2Fo-Fc electron density map (blue 1.2σ) of Axin2_361–368_. SIAH1 is represented in cyan, Axin2 is represented in purple. (**B**) Axin2_361–368_ fits into the deep groove of SIAH1 as shown on the electrostatic surface.

In the SIAH1-SBD, β1 (154–159aa), β2 (162–168aa), and β3(177–184aa) formed a deep groove for a binding substrate, and Axin2 bound to the groove in the form of a loop. Axin2 peptide_361–368_, which included the VxP motif, was visible in our electron density map, and fit into the groove (Figure 4A,B). The structure of the SIAH1 in our Axin2/SIAH1 complex was almost identical to the previously reported structure of SIAH1 [23], with a root mean square deviation (r.m.s.d) of Cα’s of 0.526 Å, indicating that the binding of Axin2 to SIAH1 does not alter its overall conformation.

SIAH1 binds to the VxP motif of Axin2′s GSK3β-binding domain, which was in agreement with Axin1 [23] and other VxP-motif-containing substrates [37]. The mass spectrometry assay found that Axin2 could immunoprecipitate with E3 ubiquitin ligase SIAH1 and SIAH2, and overexpression of wild-type SIAH1 and SIAH2, but not their RING domain mutants, decreased endogenous levels of Axin2 [23]. These results showed that the recognition patterns of SIAH1-Axin1 and SIAH1-Axin2 were almost the same, so SIAH could regulate the cytoplasmic levels of Axin2 in the same way as that of Axin1.

### 3.3. The Axin2/SIAH1 Interface and Mutagenesis Analysis

**Figure 5:**
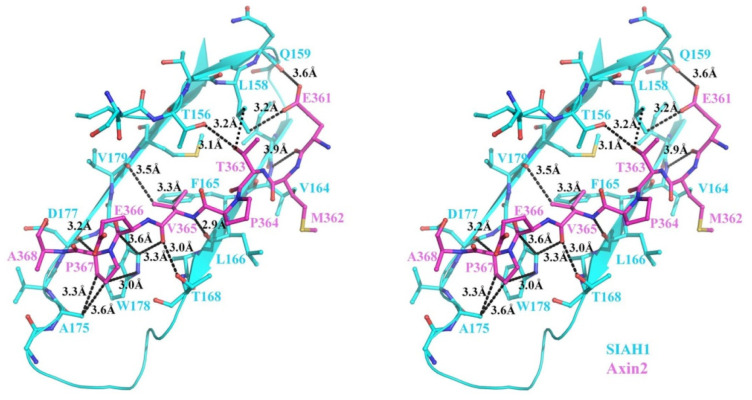
Wall-eyed view showing the critical residues in the Axin2/SIAH1 interface. SIAH1 is shown in cyan, Axin2 in purple, and the interacting amino acid residues are connected by black dotted lines representing the distance.

Axin2-GBD interacted with SIAH1-SBD mainly through hydrogen and hydrophobic bonds. Specifically, from Glu361 to A368 of the Axin2 peptide _361_**EMTPVEPA**_368_, Glu361 and Thr363 underwent a hydrogen bond with Gln159 and Thr156 on the β1 of SIAH1-SBD, respectively. Phe165, Leu166, Ala175, and Trp178 of SIAH1-SBD formed a hydrophobic pocket that interacted with a VxP motif, including Val365, Glu366, and P367 of Axin1-GBD (Figure 5).

**Figure 6:**
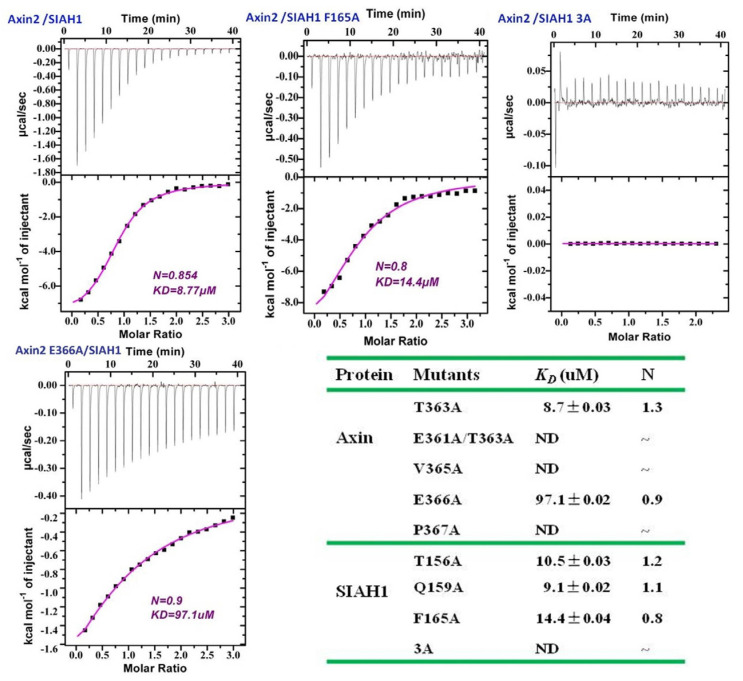
Binding affinities of Axin2 mutants with wt Axin2_356−368_ or wt SIAH1−SBD with SIAH1−SBD mutants or Axin2_356−368_ mutants measured by ITC200 assays. The K*_D_* value of wt Axin2_356−368_/wt SIAH1−SBD is 8.77 μM. ND: no detectable binding.

To assess the significance of the individual interactions observed in our structure, we mutated the key residues in Axin2_356–368_ and SIAH1-SBD to alanine and used ITC to assay their binding affinities. The results showed that the binding molar ratio of WT Axin2 and WT SIAH1 was 1:1, with a *K_D_* of 8.77μM (Figure 6). We tested the Axin2 mutants on the binding of SIAH1. A double-mutant D361A/T363A strongly reduced the binding of Axin2 to SIAH1, whereas single mutants had a smaller effect (Figure 6). This is consistent with our crystal structure which shows that Glu361 and Thr363 form hydrogen-bond interactions with SIAH1 Gln159 and Thr156, respectively (Figure 5). In addition, the single alanine substitution of the middle residue of the Axin2 VxP motif, E366A, strongly reduced the binding of Axin2 to SIAH1 with a *K_D_* of 97.1μM, while the single V365A and P367A completely abolished the interaction (Figure 6), consistent with our crystal structure which showed that Val365 and Pro367 bind to the hydrophobic core of SIAH1 rather than E366 (Figure 4B). We also tested the effect of SIAH1 mutants on the binding of Axin2. SIAH1 one-site mutants F165A decreased their affinities with Axin2 slightly, with a *K_D_* of 14.4μM, while a triple-mutant V176A/D177A/W178A blocked the ability of SIAH1 to interact with Axin2, in contrast to Thr156, Leu158, and Gln159 which did not. This is consistent with our crystal structure which revealed multiple hydrophobic interactions between SIAH1 Trp178 and Axin2 Val365 and Pro366 (Figure 5), ascribing a key role to this tryptophan residue of SIAH1 in the Axin2 and SIAH1 interaction.

### 3.4. Axin2/SIAH1 Interaction Indicates a Promising Drug-Binding Site

**Figure 7:**
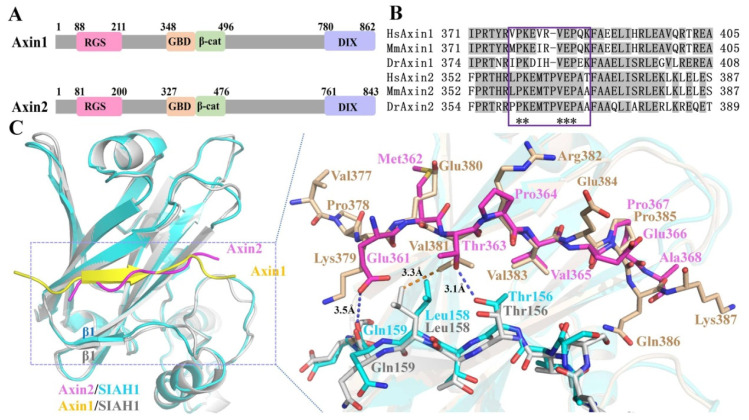
Structural comparisons of Axin2/SIAH1 and Axin1/SIAH. (**A**) Schematic diagram of the human Axin1 and Axin2 conserved domains. (**B**) Sequence alignment of the conserved VxP motifs in Axin2 and Axin1, where the highly conserved amino acids around the VxP motif and the VxP motif are shown with asterisks. Abbreviations: Hs, *Homo sapiens*; Mm, *Mus musculus*; Dr, *Danio rerio*. (**C**) Residues involved in interface interactions between the Axin1 or Axin2 and SIAH1. Axin2/SIAH1 is represented in purple and cyan, Axin1/SIAH1 is represented in yellow and gray.

Axin1 and Axin2 share more than 40% identity in amino acid sequence. They both have the GBD domain and DIX domain, as well as binding sites for SIAH, β-catenin, GSK, and other important proteins in the Wnt signaling pathway (Figure 7A). It is important to uncover the structural basis of the interaction between Axin and SIAH for the development of highly effective drug targets.

Even though Axin2 has a VxP motif similar to Axin1 in its GBD, the amino acid sequence around the VxP motif is different from Axin1 (Figure 7B). Which implies the interactions between Axin2 and SIAH1 might be distinct from Axin1/SIAH1. We carried out the structural alignment of Axin2/SIAH1 with Axin1/SIAH1 in PyMOL. Our comparison showed that SIAH1-SBD in the two structures was similar, with r.m.s.d = 0.562 Å. However, there was one marked conformational difference for the β1 of SIAH1 (residues 154–159aa): Thr156 and Gln159 within the β-sheet moved about 1.2 Å, close to the bound Axin2 (Figure 7C).

Axin2 and Axin1 fit into the same groove among SIAH1 β1, β2, and β3-sheets, but the relative positions of interacting residues were not the same. Axin2 Glu361 was at the same position of Axin1 Lys371, which formed a hydrogen-bond interaction with Gln159 of SIAH1 β1. The Axin2 hydrophilic amino acid Thr363 was at the same position of the Axin1 hydrophobic amino acid Val381, and also formed a hydrogen-bond interaction with Gln159 of SIAH1 β1. These interaction forces were different from the hydrophobic force between Axin1 Val381 and SIAH1 Leu158, and made the SIAH1 β1 shift close to the Axin2 (Figure 7C). Notably, compared with Axin1, the N-terminal of the Axin2 VxP motif had a conserved proline residue. Axin2 Pro364 had no interaction with its surrounding SIAH1 residues, but it could change the conformation of the peptide backbone, making it from the β-strand of Axin1 to the loop of Axin2 (Figure 7C). This binding mode was also different from Axin binding GSK3β which had α-helix conformation [30]. We know that if there is a drug candidate that inhibits the binding between SIAH1 and Axin2, then it may also affect the binding between SIAH1 and Axin1, or between Axin1/2 and GSK3. These can lead to many unforeseen consequences for Wnt signaling and cancer development. Hence, specialized inhibitors are particularly important. The conformational changes in the intrinsically disordered regions of Axin represent different preferences for their binding partners. We can exploit the preferences induced by conformational changes to develop small molecules or peptides that intervene in a specific binding state without affecting the binding of the target protein to another partner or its homologues to the same partner.

## 4. Conclusions

Using GST pull-down assays, we verified that Axin2-GBD was sufficient for SIAH1 binding. The crystal structure of the Axin2/SIAH1 complex was determined at 2.53 Å resolution. Based on the structural analysis combined with biochemical assays, it was revealed that one SIAH1 interacts with one Axin2 molecule. A highly conserved peptide _361_**EMTPVEPA**_368_ within Axin2-GBD binds to a deep groove formed by β1(154–159aa), β2(162–169aa), and β3(175–182aa) of SIAH1, mainly through its N-terminal hydrophilic amino acids Arg361 and Thr363 and C-terminal VxP motif. However, unlike the β-sheet of Axin1, Axin2 displayed a loop, which was a novel binding mode of SIAH and Axin. The amino acid sequence around the VxP motif was different from that of Axin1, resulting in a special interaction pattern between Axin2 and SIAH1. Wnt-induced Axin2 is a critical negative feedback regulator of the Wnt signaling pathway. This work explains the pattern of Axin2 recognition by SIAH1, complementing the role of Axin in Wnt signaling regulation, and may lay the structural foundation for the design of SIAH1 inhibitors. Whether the inhibitor could be used to treat cancer with aberrant Wnt signaling, should be investigated.

## Figures and Tables

**Table 1:** X-ray data collection and refinement statistics.

Data Collection	Axin2_356–376_/SIAH1-SBD
Space group	P2_1_
Cell dimensions	
a, b, c (Å)	40.82, 87.93, 59.70
α, β, γ (°)	90.00, 101.57, 90.00
Wavelength (Å)	0.97929
Resolution (Å)	29.2–2.53 (2.59–2.53)
R_pim_ (%)	4.6 (24.1)
CC1/2	98.3 (95.3)
Completeness (%)	99.5 (100.0)
Redundancy	6.7 (6.8)
**Refinement**	
Resolution (Å)	29.24–2.53
No. reflections	13,789
^a^ Rwork/^b^ Rfree (%)	20.68/23.53
No. atoms	3049
R.m.s deviations	
Bond lengths (Å)	0.004
Bond angles (°)	0.762
Ramachandran plot (%)	
Most favorable	92.7
Allowed	7.3
Outliers	0

Values in parentheses are for highest-resolution shell. ^a^ *R*_work_ = Σ_hkl_ |Fo(hkl) − Fc(hkl)|/Σ_hkl_ Fo(hkl). ^b^ *R*_free_ was calculated for a test set of reflections (5%) omitted from the refinement.

## Data Availability

The crystal structure presented in this study is publicly available at the Protein Data Bank under the PDB code 8HE0.
